# How does emotion influence the creativity evaluation of exogenous alternative ideas?

**DOI:** 10.1371/journal.pone.0219298

**Published:** 2019-07-05

**Authors:** Serena Mastria, Sergio Agnoli, Giovanni Emanuele Corazza

**Affiliations:** 1 Department of Electrical, Electronic, and Information Engineering “Guglielmo Marconi”, University of Bologna, Bologna, Italy; 2 Marconi Institute for Creativity (MIC), Villa Griffone, Sasso Marconi, Italy; Victoria University of Wellington, NEW ZEALAND

## Abstract

The interaction of emotions with creative cognition is one of the most intriguing topics in the creativity research. In this study, we investigated the extent to which various emotional states influence the evaluation of ideas, which is a crucial component of the creative thinking process. To this end, we used emotional (both positive and negative) and neutral pictures to induce emotional states and then asked participants to evaluate the creativity of exogenous ideas (i.e., those generated by other people) as part of an alternative use evaluation task. As the results of previous studies suggest the existence of a negative bias when judging highly creative ideas, we presented the participants with non-creative, moderately creative, and highly creative uses for everyday objects. Overall, the participants gave higher creativity ratings when under positive emotional engagement than when in negative or neutral conditions. Moreover, neutral and emotional context differently moderated the creativity evaluation of the three object use categories. Specifically, participants gave higher ratings for non-creative uses, and (to a lesser extent) for highly creative uses when in a positive emotional state, than they did when in the neutral condition. On the other hand, when in a negative emotional state, the participants gave lower ratings for moderately creative uses than they did in either the positive or neutral conditions. These data provide initial evidence that emotional states can influence the creativity evaluation of exogenous alternative ideas that are generated through divergent thinking.

## Introduction

A growing body of research is concerned with the relationship between cognition and emotion. Researchers have formulated many models to unravel the nature of this relationship [1–4]. For instance, some have attempted to understand the extent to which the cognitive evaluation of environmental events can trigger affective reactions [5–9]. Among cognitive processes, creative cognition involves specific cognitive abilities and is one of the most complex ensembles of mental functions [10]. To ensure survival, personal growth, and professional success, people need to adapt to environmental changes by adopting new problem-solving strategies, by generating potentially novel/original and appropriate/effective ideas, and by evaluating and implementing new products or solutions [11–17]. The role of emotion on creative cognition is critical and emerging as topic in the research on creativity [18, 19], as everyday emotional states can influence ideas that come to mind, and thereby influence the decision that rely on those ideas [20–22].

Several researchers have investigated the emotion-creativity relationship by using a broad range of induction procedures (e.g., emotional imagery, affective stimuli, or a combination of emotionally stimulating materials) to engage participants in the intended affective states, using various measures of creativity (see [23] for a meta-analysis). The results from some of these studies reveal that positive affective states, as compared to neutral conditions, enhance performance in divergent thinking (i.e., fluency, originality, flexibility), categorization (i.e., cognitive flexibility), remote associates test, and insight problem-solving tasks, as individuals tend to make richer associations between knowledge frames when in a positive affective state than when in a neutral state [20, 24–28]. On the other hand, some contradictory results exist regarding the role that negative emotional states play in creative thinking. Some researchers have found no difference between the negative and neutral conditions, and others have found negative affective states to have only a slightly detrimental influence on creativity, as compared to the neutral states [29–31]. In fact, some researchers have found that negative states can actually lead to stronger creative performance, as compared to neutral states [32, 33]. A similar contradictory view of the emotion-creativity link has also emerged in direct comparisons of the positive and negative affective states: whereas some studies displayed that positive affective states promoted creativity, as compared to negative states [24, 26], others showed the opposite effect [34–36]. Further studies suggested that it is the intensity of the emotional experience measured with both positive and negative high-arousal emotional states than can facilitate creativity, as compared to low-arousal states ([35, 37]; see [23] and [38] for reviews). In line with these findings, it has been shown that highly arousing situations (including negative ones such as creative frustration) can even booster creative performance in individuals with specific personality traits [39]. Although it is not still completely clear the extent to which positive and negative affective states facilitate or inhibit various facets of creativity–or how they do so–researchers are largely in agreement that emotion modulates the various components of creative thinking [19].

The lack of consistency in the literature is partly due to the multi-componential nature of the creative thinking process [40, 41], which has been described as an ensemble of components whose interaction can lead to potentially original/novel and effective/appropriate products [13, 42, 43]. The strength and direction of the effects of emotions on creative performance may vary as a function of the type of creative components involved, as well as in relation to the methodology used to measure them [23]. When reduced in terms of complexity, creative thinking can be described as the result of various processes. According to the dual-process models [40, 41], in particular, the two main components of creative thinking are generation and evaluation [44–48]. This view is consistent with the empirical results, which show that the abilities of generation and evaluation are relatively independent [49].

In this study, we specifically focused on how the evaluation of ideas is connected to emotions. Technically, the evaluation of a creative idea requires the extraction of non-obvious values, which can then be translated into a creativity score [50, 51], using more or less rigorous approaches [40, 52–55]. The evaluation of ideas can be measured as a general evaluative ability [56–58], which extends beyond evaluations of one’s own ideas [59]. Indeed, the evaluation of creativity involves not only the assessment of one’s own (endogenous) ideas but also of others’ (exogenous) ideas [52]. As other researchers have done [59–61], we asked participants to evaluate a predefined set of exogenous ideas because the judgments of their own ideas could be biased ([52, 59]; see also [62]).

Although researchers have dedicated extensive empirical work to how emotions affect the generation of ideas, they have scarcely examined the ways in which emotions affect evaluations of creativity. To our knowledge, researchers have explored emotion’s influence on the recognition of creativity in only two recent seminal studies [63, 64]. These studies have specifically focused on uncertainty, which seems to facilitate negative attitudes or implicit bias against creativity [64]. The results of these studies also suggest the existence of ambivalent attitudes towards creative evaluation, as the participants who experienced low levels of uncertainty (e.g., anger and happiness) had positive attitudes towards creative evaluation, whereas the participants who experienced high uncertainty (e.g., fear) exhibited negative biases regarding the recognition of creativity [63, 64]. However, in these studies, the researchers experimentally manipulated emotions by focusing on valence [63], rather than arousal.

In this study, we systematically explored how both positive and negative emotions (which we balanced in terms of arousal) affect the evaluation of exogenous ideas. We hypothesized, based on previous results, that individual’s emotional states would modulate their abilities to judge the creativity of exogenous ideas. We positioned this investigation within a theoretical framework in which creativity is viewed as a dynamic phenomenon, involving emotional states, socio-cultural effects, and complex, time-dependent system interactions [65–68]. More specifically, according to the dynamic definition of creativity [13, 69], the evaluation of ideas that are generated as outcomes of a creative thinking process cannot be interpreted as an absolute judgment; rather, it is an exercise in estimation–an active interaction between the evaluator and the product–that is dependent on the evaluator’s emotional state [70]. A thorough understanding of the impact of emotional states of judges and of the interaction of these with the item to be assessed comes as a straightforward necessity. This work aims at providing a step forward in this direction.

### The research problem

Our aim in this study is to investigate whether and to which extent the participants’ emotional experiences can modulate the creativity evaluation of exogenous ideas representing alternative uses for everyday objects. To this purpose, we induced positive, negative, and neutral emotional states in the participants through evocative pictures–controlling for the level of arousal–and contrasted each of the positive and negative conditions with the neutral condition. We embraced the widely accepted view adopted from the emotion-creativity research involving self-report and psychophysiological responses that emotional states can be differentiated in terms of their hedonic tone (positive vs negative) and by the extent to which they arouse and activate (low *vs*. high, [71–73], see [74] for a meta-analysis). During emotional engagement, we asked the participants to perform the Alternative Use evaluation Task (AUeT), which involved viewing exogenous ideas involving uses for everyday objects. We had classified these uses as non-creative (NC; e.g., a bicycle as a means of transport), moderately creative (MC; e.g., a bicycle as a slide), and highly creative (HC; e.g., a bicycle as a chandelier). The participants evaluated these ideas’ creativity on a trial-by-trial basis (see [75] for a similar procedure).

#### Research question 1: Can emotions influence the evaluation of exogenous ideas?

In this study, we examined whether emotions impact the evaluation of others’ ideas. According to the creativity literature dedicated to emotions affecting diverse components of creative thinking, including idea generation and evaluation, some general predictions can be formulated. Assuming that positive emotional states inform individuals that their environments are benign, thus inducing risk-taking tendencies [76, 77] and facilitating performance on diverse measures of creativity [78, 79], then such positive emotions could also help recognizing the positive aspects of the ideas. Individuals in such positive states may thus be more inclined than those in neutral states to detect the quality of others’ ideas, which would result in higher scores in judgments of creativity. On the other hand, assuming that negative emotional states inform individuals that their environments are problematic, thus inducing risk aversion [80] and causing adverse effects in terms of creative fluency and flexibility (e.g., in ideation, [40]), then such negative emotions could also lead to more severe evaluations of ideas. Individuals in such negative states may thus be less inclined than those in neutral states to accept novel solutions and creative ideas, which would result in lower scores in judgments of creativity. It has been indeed demonstrated that individuals who experience negative states (e.g., fear) tend to produce lower creativity ratings because of implicit biases against creativity [63, 64]. This view would be in line with the *hedonic-tone hypothesis*, which assumes that positive and negative states have differential influences on creativity: Positive emotional states should facilitate creative performance, and negative emotional states should inhibit it [28, 81].

On the other hand, if highly arousing emotional states (both positive and negative) increase the participants’ overall capacity to perceive, process, and evaluate information, thus promoting innovative responses [38, 82–84], then high arousal should facilitate more indulgent evaluations of ideas, as compared to low arousal (i.e., the neutral condition). In other words, individuals who experience high arousal are usually more inclined than those with low arousal to include new cognitive categories, combine information, and consider multiple novel alternatives [11, 38], so they might also be more better able to detect value in new ideas and to thus overestimate those ideas’ creativity. This view would support the *activating hypothesis*, which claims that high arousal is associated to higher creativity than low arousal: According to this hypothesis, both positive and negative states facilitate performance on creativity tasks because of their activating nature [11, 38, 85].

#### Research question 2: Can emotions influence the evaluation of exogenous ideas differently at varying levels of idea creativity?

To examine the extent to which emotions influence the evaluation of others’ idea, we considered the relationship between the evaluator’s emotional experience and the nature of the object use (in terms of the creativity level), as expert raters had previously judged (as NC vs. MC vs. HC). Based on the results of previous studies, we also predicted a negative bias in judgments of creative ideas [63, 64] according to the idea that greater novelty leads to greater uncertainty regarding whether an idea is reliably effective or workable [86–88]. This feeling of uncertainty seems to activate negative associations with creativity, which in turn can result in lower evaluations of creative ideas [64]. “Bias against creativity” might play a role in the idea evaluation process and this is more likely to occur in highly creative ideas when people experience an aversive state than positive or neutral state. Accordingly, we expected that NC ideas (i.e., those that are totally lacking in novelty) would be less sensitive than MC or HC to the negative emotional states. On the contrary, we expected that MC and HC ideas, due to their novelty, would be more sensitive to negative emotional states than to positive and neutral ones. All in all, testing these hypotheses provides further insight into the mechanisms underlying the influence of evaluators’ emotional experience on creativity judgments of others’ ideas as a function of the nature of the idea.

## Method

### Ethics statement

The study conformed to the Declaration of Helsinki and was approved by the Ethical Committee of the Department of Psychology at the University of Bologna. We obtained written, informed consent from all participants.

### Participants

We calculated the sample size that was necessary to achieve 95% power (which is required to detect an adequate effect) a-priori using G*power software, version 3.1 [89]. Based on *f* = 0.4 [90], this power calculation yielded a recommended sample size of 48 participants. We thus required 55 adult students from the University of Bologna (33 females, *M*_*age*_ = 26.07, *SD* = 5.34) to participate in the study. All participants had normal or corrected-to-normal vision, and none of them reported current or past neurological or psychopathological problems. None of the participants had previous experience with the materials used in this experiment, and we paid each participant 10 euros as compensation.

### Stimuli and apparatus

We presented the participants with images from a set of 252 colour pictures of natural scenes so as to induce positive, neutral or negative emotional states. We selected these images from the International Affective Picture System (IAPS, [91]; see S1 File in the Supporting Information), as well as public-domain pictures from the Internet. The images’ contexts are positive (e.g., pictures of erotic or romantic couples), neutral (e.g., portraits or images of multiple people in an everyday context), or negative (e.g., photos of mutilated bodies or violence). We balanced these emotional categories and ensured that they did not differ in terms of arousal, based on the results of a previous psychophysiological study [92]. We uniformly allocated the picture exemplars such that there were 84 images in each of the positive, neutral, and negative categories. Each image subtended a visual angle of 10.2° (width) × 5.8° (height), at a constant viewing distance of 100 cm. We also balanced all pictures for contrast and brightness (0.1 and 0.6 on a linear scale from 0 to 1).

We derived this study’s AUeT stimulus materials from a database in a previous study [93], in which 40 participants generated 3720 uses for 20 objects during AUT. Two expert raters originally evaluated the creativity of these responses (inter-rater agreement Cohen's κ = 0.65) on a scale from 1 to 5 [54, 94]. For the purpose of this study, we defined the three object-use categories in terms of creativity level (NC, MC, and HC), based on the judgement scale of Silvia et al. [95]. As a general rule, NC corresponds to a consensus assessment equal to 1, MC corresponds to 3, and HC corresponds to 5. However, the number of alternative uses associated with the highest score was very limited–corresponding to the 0,7% of all responses–because of the highly skewed distribution in creative production [96], ratings of 4 were included in the HC category to ensure an adequate number of stimuli in that category. It is worth highlighting that the uses with scores of 4 were above the 95th percentile in terms of overall creative production. Moreover, we used the following stimuli-selection criteria: the numbers of words in describing each object-use description and the number of uses for each object (at least seven) across the three levels of creativity. These criteria allowed us to control for the variability in the lengths of the object-use descriptions and in the number uses within each level. The final set of stimuli consisted of 252 uses (84 each for NC, MC, and HC), divided evenly among 12 objects. We presented each use to the participants in the verbal form (descriptor length *M* = 3.15 words, *SD* = 1.09) along with a corresponding colour image of the associated everyday object (1.89° horizontal × 1.89° vertical visual angle). We presented all visual stimuli on a 19-inch LCD monitor with1024 × 768 pixel resolution and a 60 Hz refresh rate, using a constant aspect ratio. Stimuli were presented electronically using the E-Prime 3.0 software [97], and behavioural responses were collected using a standard computer keyboard. Please refer to S2 File in the Supporting Information for the full list of AUeT stimuli (including uses and corresponding objects).

### Experimental design and procedure

Upon their arrival, we informed the participants about the experimental procedures. After which, they signed consent forms. Before beginning the experiment, we conducted five practice trials to familiarize the participants with the experimental design. The participants then completed the AUeT [75]. In each trial, they saw a colour image of an object in the centre of the screen, paired with a verbal description of a use related to that object, (at the bottom of the picture of the object). We asked the participants to rate the creativity of each use on a 5-point-scale ranging from 1 (*not at all creative*) to 5 (*very creative*). Before they began the AUeT, we explicitly instructed the participants in the use of the rating scale and explained the criteria using the instructions for judging creativity described in Silvia et al. [95].

To induce different emotional states in participants during the object-use evaluation, we used IAPS pictures [91], as they originate from a well-controlled stimulus set that is frequently used in emotion research. Based on the overall ratings for valence and arousal of the affective space [98], the photographs of erotic scenes or romantic couples induced a pleasant affect (appetitive motivation), and images of mutilations or violent scenarios induced an unpleasant affect (defensive motivation) [99]. We explicitly told the participants that these pictures, which preceded the object presentation, were irrelevant to the task and thus should to be ignored. The experiment comprised 252 trials. We presented the IAPS images in six blocks of 42 pictures: two blocks each of positive, neutral, and negative pictures. We showed each stimulus category equivalently in the first and the second halves of the experiment, using two distinct orders, counterbalanced across participants. There was a 6-min pause between blocks. After each block, the participants rated their current emotional states by using the most common measure of the two-dimensional affective domain, i.e., the Positive and Negative Affect Scale (PANAS; [100], see also [71, 98, 101–106]).

**Fig 1:**
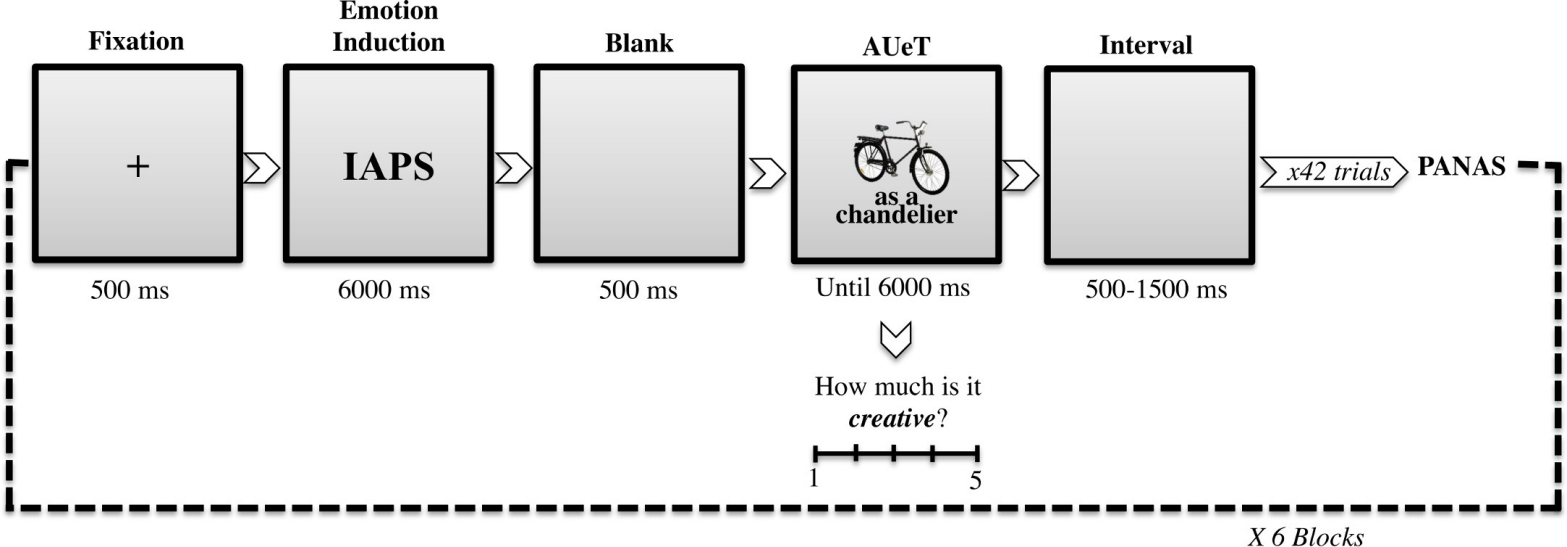
Overview of the trial procedure. Example of trial structure in wich an IAPS image is presented before the AUeT.

Each block included an equal number of NC, MC, and HC uses for objects (14 trials per condition in each of the six blocks). We pseudo-randomized the order of the object uses, with the constraint that no object or use category could occur more than twice consecutively. As depicted in Fig 1, in each trial, after a fixation cross appeared at the centre of the screen for 500 ms, a natural scene (from IAPS) was visible for 6000 ms. Then, after a blank screen (500 ms), a picture of an object and a verbal description of a proposed use were shown until the participant evaluated the creativity of that use using the 5-point scale by hitting the appropriate number on the keyboard. We provided no instructions regarding the response speed. We then provided a randomly set variable interval of 500 to 1500 ms before the start of the next trial.

### Data analysis

In order to check the effect of EMOTIONAL CONTEXT (3 within-subjects levels: positive/appetitive, neutral, negative/aversive) on the participants’ affect self-assessments, two distinct generalized linear mixed models (AR1 covariance structure) were performed on the positive- and negative-affect PANAS dimensions, respectively. Robust error estimation was used to control for the effect of outliers [107].

Crucially for the research question that inspired this work, participant’s evaluation scores to noncreative, moderately creative, and highly creative object use categories were explored in a generalized linear mixed model (AR1 covariance structure), with robust error estimation, as a function of emotional context. A 3 (EMOTIONAL CONTEXT: positive, neutral, negative) X 3 (CREATIVITY LEVEL: NC, MC, and HC) within-subjects design was tested exploring main and interaction effects.

## Results

### Manipulation check for the induction of emotion

The results demonstrated the effectiveness of the emotional induction on participants’ affect self-assessments. Significant differences emerged between the three induced emotional states (positive, neutral, and negative) on PANAS positive affect, *F*(2,162) = 11.57, *p* < 0.001, as well as on PANAS negative affect, *F*(2,162) = 28.07, *p* < 0.001. Specifically, the participants’ self-assessments of PANAS positive affect dimension were significantly more positive in the positive emotional condition than those in the neutral condition (*b* = 0.19, *t*_*324*_ = 3.317, *p* = 0.001, 95% CI = [0.077, 0.303]), or the negative emotional condition (*b* = 0.28, *t*_*324*_ = 4.703, *p* = 0.000, 95% CI = [0.162, 0.398]). On the other hand, the participants’ self-reports of PANAS negative affect dimension were significantly more negative in the negative emotional condition than those in the neutral condition (*b* = - 0.59, *t*_*324*_ = - 7.105, *p* = 0.000, 95% CI = [-0.760, -0.429]), or the positive emotional condition (*b* = - 0.59, *t*_*324*_ = - 7.487, *p* = 0.000, 95% CI = [-0.754, -0.439]).

### Emotion’s effects on the evaluation of ideas

**Fig 2:**
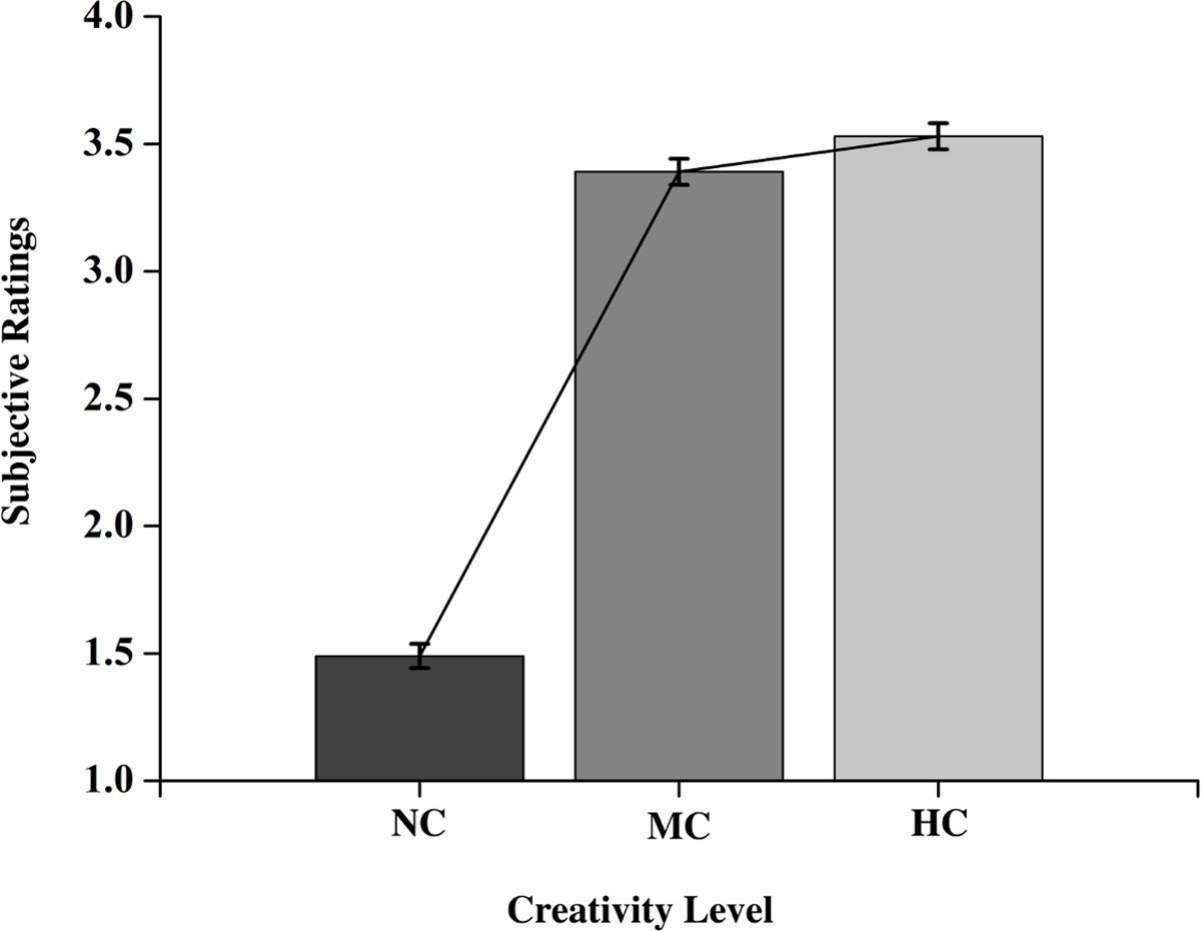
Evaluation of ideas. Average subjective ratings as a function of non-creative (NC), moderately creative (MC), and highly creative (HC) object uses. The error bars indicate 1 SEM.

We observed two clear main effects. The first, an expected CREATIVITY LEVEL main effect, *F*(2,486) = 130.93, *p* < 0.001, indicated that participants evaluated the NC uses as less creative than both the MC (*b* = - 1.90, *t*_*486*_ = - 15.454, *p* = 0.000, 95% CI = [-2.142, -1.659]) and the HC (*b* = - 2.04, *t*_*486*_ = - 16.038, *p* = 0.000, 95% CI = [-2.290, -1.790]) uses. The ratings for MC uses were also lower than those for HC uses (*b* = - 0.140, *t*_*486*_ = - 6.547, *p* = 0.000, 95% CI = [-0.182, -0.098]; see Fig 2).

**Fig 3:**
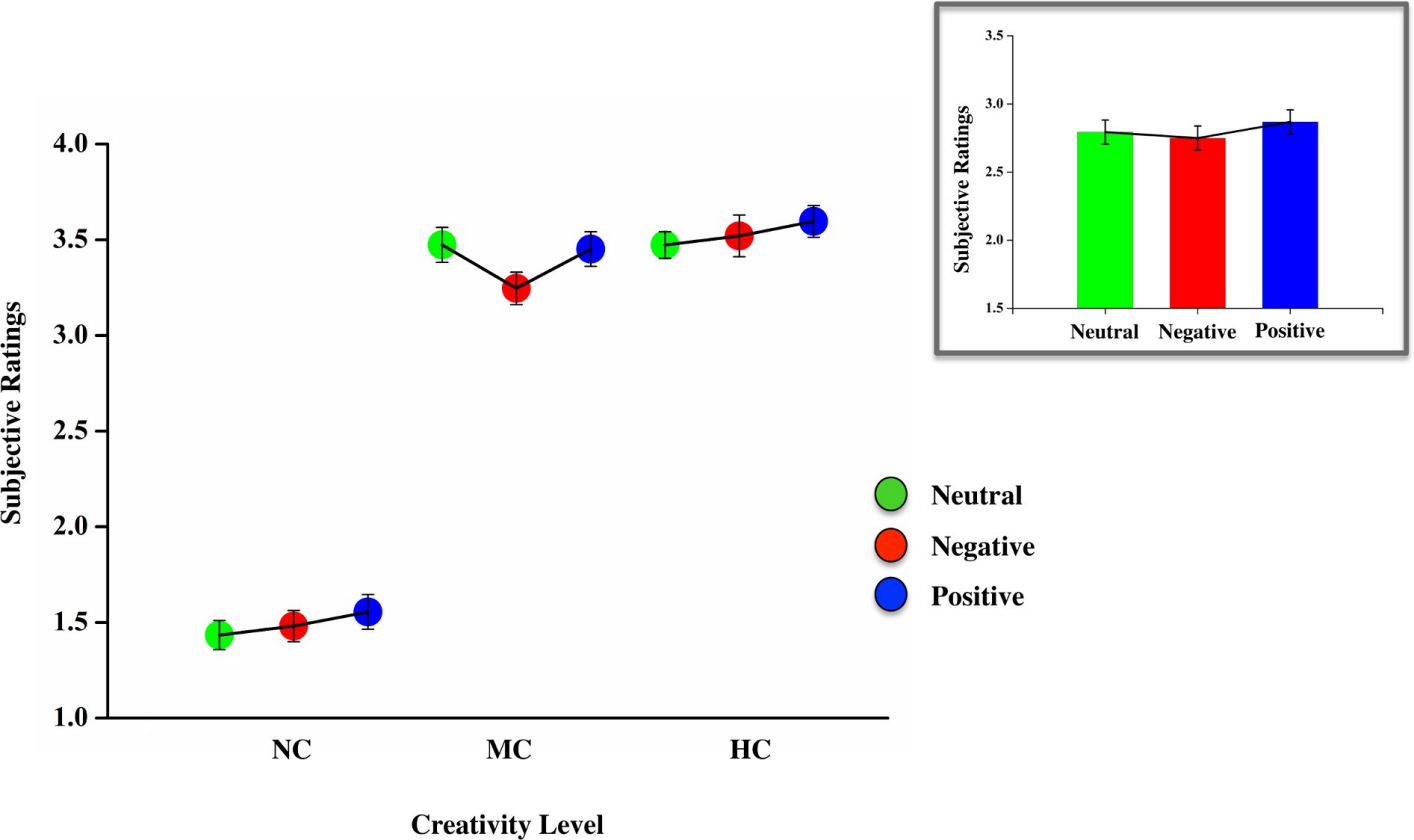
Emotion’s effects on the evaluation of ideas. Average subjective ratings as a function of the non-creative (NC), moderately creative (MC), and highly creative (HC) object use categories in the neutral, negative and positive emotional contexts. The insert shows the overall effect that neutral, negative, and positive emotional contexts have on evaluations of creativity. The error bars indicate 1 SEM.

The second main effect was of EMOTIONAL CONTEXT, *F*(2,486) = 12.13, *p* < 0.001, as the creativity scores were higher overall in the positive emotional context than in either the neutral (*b* = 0.074, *t*_*486*_ = 2.469, *p* = 0.014, 95% CI = [0.015, 0.133]) or negative (*b* = 0.119, *t*_*486*_ = 4.927, *p* = 0.000, 95% CI = [0.071, 0.166]) conditions: the latter two conditions did not differ significantly (*b* = 0.044, *t*_*486*_ = 1.607, *p* = 0.109, 95% CI = [-0.010, 0.099]; see the insert in Fig 3). Interestingly, we also observed a significant interaction between CREATIVITY LEVEL and EMOTIONAL CONTEXT, *F*(2,486) = 9.88, *p* < 0.001, which indicates that the influence of the emotional states changes as a function of the creativity level (see Fig 3).

Specifically, in evaluating NC uses, the positive emotional states enhanced the rating scores than either the neutral (*b* = 0.121, *t*_*486*_ = 3.058, *p* = 0.002, 95% CI = [0.043, 0.199]), or negative (*b* = 0.074, *t*_*486*_ = 2.289, *p* = 0.022, 95% CI = [0.010, 0.138]) conditions,; the latter two did not differ significantly (*b* = -0.047, *t*_*486*_ = -1.640, *p* = 0.102, 95% CI = [-0.104, 0.009]). In the evaluations of MC uses, however, the induction of a negative emotional context lead to lower ratings than either the neutral (*b* = - 0.228, *t*_*486*_ = - 4.297, *p* = 0.000, 95% CI = [-0.332, -0.124]) or the positive (*b* = - 0.206, *t*_*486*_ = - 4.031, *p* = 0.000, 95% CI = [-0.306, -0.106]), conditions; again the latter two did not differ significantly (*b* = 0.022, *t*_*486*_ = 0.423, *p* = 0.672, 95% CI = [-0.080, 0.125]). Finally, the evaluation scores for the HC uses showed significant differences between the positive and the neutral conditions (*b* = 0.123, *t*_*486*_ = 2.354, *p* = 0.019, 95% CI = [0.020, 0.226]), but not between either the positive and negative conditions (*b* = 0.076, *t*_*486*_ = 1.649, *p* = 0.100, 95% CI = [-0.015, -0.020]), or the neutral and negative conditions (*b* = -0.047, *t*_*486*_ = -0.658, *p* = 0.511, 95% CI = [-0.189, 0.094]).

## Discussion

As previously stated, a critical question in the research on creativity regards the extent to which emotions can affect the generative and evaluative components of creative thinking [40–44]. For this study, we considered the process of evaluating creativity as a general ability to evaluate exogenous ideas [56–58], independent from the ability to evaluate endogenous ideas [59]. The present study provided an initial exploration of the impact of emotions, as engaged by positive and negative visual stimuli, on creativity evaluation of exogenous ideas generated during an alternative use task (AUT). We used in particular an alternative use evaluation task (AUeT), by asking participants to evaluate ideas, which represented noncreative, moderately creative, or highly creative uses for everyday objects.

Results reveal that the participants who were in a positive state, as compared to those in a negative state or neutral (control) state, tended to be more indulgent when estimating the creativity of the ideas. These findings appear to be consistent with certain past findings on the relationship between moods and creativity, even if referred to standard indicators of creative ideation, problem-solving [26, 76, 108] and divergent thinking abilities [38, 109]. Similarly, we observed that a positive emotional state, as compared to a negative or neutral state, facilitated evaluation ability. These findings are in line with the hedonic-tone hypothesis, according to which positive hedonic states, unlike negative or neutral ones, inform individuals about safety of the surrounding environment and thus are associated to tendencies towards over inclusion and loose conceptual boundaries [110]. Positive emotional states thereby promote individuals ‘exploration of novel ideas and creative solutions [78, 79]. In the same way, we found that these tendencies influenced the creativity evaluations of alternative ideas, likely promoting individuals to extract more value during the positive-state assessments than during the negative or neutral state, thus increasing their scores.

In addition, we assessed the extent to which positive and negative emotional states affect the process of evaluating the creativity of ideas: to do so, we presented participants with various categories of object uses based on independent expert raters’ creativity ratings (as NC, MC, or HC). The results of our study partially support the prediction in which, as compared to NC uses, the evaluation of uses with varied creativity could be more sensitive to a negative emotional state more than to a positive or a neutral state, because of the feeling of uncertainty that people develop when evaluating creative ideas ([63, 64]; see also [86–88]). More specifically, we found that, although positive emotional experiences led to higher creativity for NC uses, and to a lesser extent for HC ones, as compared to the neutral condition, negative emotional experiences led to lower creativity ratings for MC uses, as compared to both the positive and neutral conditions. Thus, we hypothesize that there are two directions of emotional influence on creativity judgements as a function of the uses’ creativity.

For NC and HC uses, our data support the aforementioned hedonic-tone hypothesis, in which positive feelings promote creativity performance, including in evaluation [20, 24–28, 111, 112]. On the other hand, the data on MC uses appear to be in line with the prediction that a negative state, as compared to an positive or a neutral one, can lead to lower creativity ratings during experiences of novelty or uncertainty [86–88; 64]. In addition, these results are consistent with the general assumption that a negative affective state, which informs individuals that their situations are problematic, has an unfavourable impact on creative performance [29–31, 113]. We interpret this effect on the MC use by considering the fact that, when faced with MC ideas–possibly characterized by some form of inappropriateness [59]–people may experience feelings of uncertainty about whether those idea are reliable functional, useful, faultless, or consistent. A negative emotional state thus could inhibit the extraction of favourable values from these ideas, resulting in lower scores for creativity [63, 64, 86, 87, 114, 115]. Judging the quality of an idea in terms of appropriateness is indeed particularly difficult [49, 116], as this measure is a key element that allows individuals to distinguish between novel (or inappropriate) and creative (or appropriate) ideas [59]. In other words, a negative emotional context, unlike a positive or neutral one, may lead individuals to underestimate the creativity of MC uses, as these uses are likely to contain more uncertainty and lesser appropriateness than NC or HC. It has been indeed suggested that appropriateness reduces uncertainty, thus allowing novel or inappropriate ideas to appear to be truly creative [117].

Despite these promising findings, caution needs to be exerted when drawing conclusions from them. In the creativity judgements for NC and HC uses, the positive emotional states increased the creativity ratings for both types of stimulus, thus confirming the hedonic-tone hypothesis. However, this effect was more easily interpretable in the case of NC ideas. Specifically, the creativity judgements of NC uses were higher during positive engagement than during either neutral or negative engagement. In the case of HC uses, we found instead that creativity judgements during positive engagement were greater then those during neutral engagement but did not differ from those during negative engagement. In this case, we could not rule out the activating hypothesis, according to which both positive and negative states facilitate evaluations of creativity, as compared to the neutral condition [35, 37, 38]. One methodological implication of these results involves investigating the evaluation of ideas using categories that vary based on creativity level. Because of these results seem to suggest the importance of appropriateness in the relationship between emotion and the evaluation od ideas, future researchers could employ idea categories that are balanced for both novelty/originality and appropriateness/effectiveness [59, 118–120], in order to clarify the interaction between these dimensions and the evaluator’s emotional states.

Moreover, it may be interesting to explore the effect of emotion on evaluations of self-generated ideas. In this study, we asked the participants to judge ideas that others had generated, as measuring the judgement of individuals’ own ideas has serious methodological issues related to those individual’s bias regarding their own creative ability ([59]; see also [52]). However, the ability to assess one’s own ideas is also a critical skill in creative thinking [121–123]. Consequently, by using ad hoc experimental designs to overcome the aforementioned methodological issues, studies on how emotions impact the evaluation of endogenous ideas could provide important insights on the mechanisms underlying the selection of the most creative idea by the thinker from among a variety of alternatives that are generated during divergent thinking [70, 124].

### Conclusions

In summary, our findings provide an initial understanding of how the idea-evaluation process is moderated during affectively arousing situations. Specifically, we examined the extent to which emotional states influence the way people judge the creativity of ideas representing noncreative, moderately creative, and highly creative uses for everyday objects. Overall, results revealed that people produce higher creativity ratings when under positive emotional engagement than negative or neutral conditions. We found that, when considering the creativity level of an idea, participants gave more indulgent creativity ratings for NC and HC uses when in a positive emotional state; they underestimated MC uses when in a negative emotional state. Taken together, these findings provide the first evidence that emotions impact people’s evaluations of creativity of exogenous alternative ideas that are generated through divergent thinking.

## Supporting information

S1 File IAPS codes. (DOCX)

S2 File AUeT stimuli. (DOCX)

## References

[1] Kagan J. (1976). Emergent themes in human development. *American Scientist*. 1259233

[2] Lewis M., Sullivan M. W., & Michalson L. (1984). The cognitive-emotional fugue. *Emotions*, *cognition*, *and behavior*, 264.

[3] McCall R. B. (1972). Smiling and vocalization in infants as indices of perceptual-cognitive processes. *Merrill-Palmer Quarterly of Behavior and Development*, 18(4), 341–347.

[4] Ulvund S. (1980). Cognition and motivation in early infancy. *Human Development*, 23, 17–32. 7353876 10.1159/000272535

[5] Lazarus R. S. (1966). Psychological stress and the coping process.

[6] Lazarus R. S. (1982). Thoughts on the relations between emotion and cognition. *American psychologist*, 37(9), 1019.

[7] Lazarus R. S., Averill J. R., & Opton E. M. Jr (1970). Towards a cognitive theory of emotion In *Feelings and emotions* (pp. 207–232). Academic Press.

[8] Lazarus R. S., & Smith C. A. (1988). Knowledge and appraisal in the cognition—emotion relationship. *Cognition & Emotion*, 2(4), 281–300.

[9] Frijda N. H. (1986). *The emotions*. Cambridge University Press.

[10] Guilford J. P. (1967). Creativity: Yesterday, today and tomorrow. *The Journal of Creative Behavior*, 1(1), 3–14.

[11] Amabile T. M. (1983). The social psychology of creativity: A componential conceptualization. *Journal of personality and social psychology*, 45(2), 357.

[12] Beghetto R. A., & Kaufman J. C. (2007). Toward a broader conception of creativity: A case for" mini-c" creativity. *Psychology of Aesthetics*, *Creativity*, *and the Arts*, 1(2), 73.

[13] Corazza G. E. (2016). Potential originality and effectiveness: The dynamic definition of creativity. *Creativity Research Journal*, 28(3), 258–267.

[14] Runco M. A., & Jaeger G. J. (2012). The standard definition of creativity. *Creativity Research Journal*, 24(1), 92–96.

[15] Runco M. A. (2004). Everyone has creative potential.

[16] Simonton D. K. (2003). Scientific creativity as constrained stochastic behavior: the integration of product, person, and process perspectives. *Psychological bulletin*, 129(4), 475 12848217 10.1037/0033-2909.129.4.475

[17] Sternberg R. J., & Lubart T. I. (1999). The concept of creativity: Prospects and paradigms. *Handbook of creativity*, 1, 3–15.

[18] Mumford M. D. (2003). Where have we been, where are we going? Taking stock in creativity research. *Creativity research journal*, 15(2–3), 107–120.

[19] Kaufmann G. (2003). Expanding the mood-creativity equation. *Creativity Research Journal*, 15(2–3), 131–135.

[20] Ashby F. G., Isen A. M., & Turken A. U. (1999). A neuropsychological theory of positive affect and its influence on cognition. *Psychological Review*, 106, 529–550. 10467897 10.1037/0033-295x.106.3.529

[21] George J. M., & Brief A. P. (1996). *Motivational agendas in the workplace*: *The effects of feelings on focus of attention and work motivation*. Elsevier Science/JAI Press.

[22] Isen A. M., & Baron R. A. (1991). Positive affect as a factor in organizational behavior. *Research in Organizational Behavior*, 13, 1–53.

[23] Baas M., De Dreu C. K., & Nijstad B. A. (2008). A meta-analysis of 25 years of mood-creativity research: Hedonic tone, activation, or regulatory focus?. *Psychological bulletin*, 134(6), 779. 18954157 10.1037/a0012815

[24] Forgas J. P. (2000). *Feeling and thinking*: *The role of affect in social cognition*. Paris: Cambridge University Press.

[25] Hirt E. R. (1999). Mood In Runco M. A & Pritzker S. R (Eds.). *Encyclopedia of creativity* (Vol. 2, pp. 241–250). New York: Academic Press.

[26] Isen A. M., Daubman K. A., & Nowicki G. P. (1987). Positive affect facilitates creative problem solving. *Journal of Personality and Social Psychology*, 52(6), 1122–1131. 3598858 10.1037//0022-3514.52.6.1122

[27] Isen A. M., & Daubman K. A. (1984). The influence of positive affect on categorization. *Journal of Personality and Social Psychology*, 47(6), 1206–1217.

[28] Lyubomirsky S., King L., & Diener E. (2005). The benefits of frequent positive affect: Does happiness lead to success? *Psychological Bulletin*, 131(6), 803–855. 10.1037/0033-2909.131.6.803 16351326

[29] Goritz A. S., & Moser K. (2003). Mood and flexibility in categorization: A conceptual replication. *Perceptual and Motor Skills*, 97(1), 107–119. 10.2466/pms.2003.97.1.107 14604029

[30] Mikulincer M., Paz D., & Kedem P. (1990). Anxiety and categorization: II. Hierarchical levels of mental categories. *Personality and Individual Differences*, 11, 815–821.

[31] Verhaeghen P., Joormann J., & Khan R. (2005). Why we sing the blues: The relation between self-reflective rumination, mood, and creativity. *Emotion*, 5, 226–232. 10.1037/1528-3542.5.2.226 15982087

[32] Adaman J. E., & Blaney P. H. (1995). The effects of musical mood induction on creativity. *Journal of Creative Behavior*, 29(2), 95–108.

[33] Carlsson I. (2002). Anxiety and flexibility of defense related to high or low creativity. *Creativity Research Journal*, 14, 341–349.

[34] Bartolic E. I., Basso M. R., Schefft B. K., Glauser T., & Titanic Schefft M. (1999). Effects of experimentally induced emotional states on frontal lobe cognitive task performance. *Neuropsychologia*, 37, 677–683. 10390029 10.1016/s0028-3932(98)00123-7

[35] George J. M., & Zhou J. (2007). Dual tuning in a supportive context: Joint contributions of positive mood, negative mood, and supervisory behaviors to employee creativity. *Academy of Management Journal*, 50, 605–622.

[36] Kaufmann G., & Vosburg S. K. (2002). The effects of mood on early and late idea production. *Creativity Research Journal*, 14(3–4), 317–330.

[37] Russ S. W., & Grossman McKee A. (1990). Affective expression in children’s fantasy play, primary process thinking on the Rorschach, and divergent thinking. *Journal of Personality Assessment*, 54, 756–771. 10.1080/00223891.1990.9674036 2348355

[38] De Dreu C. K., Baas M., & Nijstad B. A. (2008). Hedonic tone and activation level in the mood-creativity link: toward a dual pathway to creativity model. *Journal of personality and social psychology*, 94(5), 739 10.1037/0022-3514.94.5.739 18444736

[39] Agnoli S., Franchin L., Rubaltelli E., & Corazza G. E. (2018). The emotionally intelligent use of attention and affective arousal under creative frustration and creative success. *Personality and Individual Differences*.

[40] Runco M. A., & Chand I. (1995). Cognition and creativity. *Educational psychology review*, 7(3), 243–267.

[41] Simonton D. K. (2013). What is a creative idea? Little-c versus Big-C creativity. *Handbook of research on creativity*, 2, 69–83.

[42] Lubart T. I. (2001). Models of the creative process: Past, present and future. *Creativity research journal*, 13(3–4), 295–308.

[43] Lubart T. I., Zenasni F., & Barbot B. (2013). Creative potential and its measurement. International Journal of Talent Development and Creativity, 1(2), 41–51.

[44] Ellamil M., Dobson C., Beeman M., & Christoff K. (2012). Evaluative and generative modes of thought during the creative process. *Neuroimage*, 59(2), 1783–1794. 10.1016/j.neuroimage.2011.08.008 21854855

[45] Finke R. A., Ward T. B., & Smith S. M. (1992). Creative cognition: Theory, research, and applications.

[46] Israeli N. (1962). Creative processes in painting. *The Journal of general psychology*, 67(2), 251–263. 13956945 10.1080/00221309.1962.9711553

[47] Wallas G. (1926). The art of thought.

[48] Allen A. P., & Thomas K. E. (2011). A dual process account of creative thinking. *Creativity Research Journal*, 23(2), 109–118.

[49] Runco M. A., & Charles R. E. (1993). Judgments of originality and appropriateness as predictors of creativity. *Personality and individual differences*, 15(5), 537–546.

[50] Boden M. (2003). *The Creative Mind*: *Myths and Mechanisms*, Routledge; 2nd edition.

[51] Howard-Jones P. A., & Murray S. (2003). Ideational productivity, focus of attention, and context. *Creativity research journal*, 15(2–3), 153–166.

[52] Runco M. A., & Smith W. R. (1992). Interpersonal and intrapersonal evaluations of creative ideas. *Personality and Individual Differences*, 13(3), 295–302.

[53] Kleinmintz O. M., Goldstein P., Mayseless N., Abecasis D., & Shamay-Tsoory S. G. (2014). Expertise in musical improvisation and creativity: The mediation of idea evaluation. *PloS one*, 9(7), e101568 10.1371/journal.pone.0101568 25010334 PMC4092035

[54] Silvia P. J. (2008). Discernment and creativity: How well can people identify their most creative ideas?. *Psychology of Aesthetics*, *Creativity*, *and the Arts*, 2(3), 139.

[55] Runco M. A. (2003). Education for creative potential. *Scandinavian Journal of Educational Research*, 47(3), 317–324.

[56] Runco, M. A., & Chand, I. (1994). Problem finding, evaluative thinking, and creativity. In Portions of this chapter were presented at the meeting of the American Psychological Assn in San Francisco, CA, Aug 1991. Ablex Publishing.

[57] Runco M. A., & Acar S. (2012). Divergent thinking as an indicator of creative potential. *Creativity Research Journal*, 24(1), 66–75.

[58] Runco M. A., & Chand I. (1994). Problem finding, evaluative thinking, and creativity In Runco M. A (Ed.), *Problem finding*, *problem solving*, *and creativity* (pp. 40–76). Norwood, NJ: Ablex.

[59] Benedek M., Nordtvedt N., Jauk E., Koschmieder C., Pretsch J., Krammer G., et al (2016). Assessment of creativity evaluation skills: A psychometric investigation in prospective teachers. *Thinking Skills and Creativity*, 21, 75–84.

[60] Rataj K., Nazareth D. S., & Van Der Velde F. (2018). Use a spoon as a spade?: Changes in the upper and lower alpha bands in evaluating alternate object use. *Frontiers in Psychology*, 9, 1941 10.3389/fpsyg.2018.01941 30405471 PMC6206077

[61] Ivancovsky T., Shamay-Tsoory S., Lee J., Morio H., & Kurman J. (2019). A dual process model of generation and evaluation: A theoretical framework to examine cross-cultural differences in the creative process. *Personality and Individual Differences*, 139, 60–68.

[62] Simonton D. K. (1988). *Scientific genius*: *A psychology of science*. Cambridge University Press.

[63] Lee Y. S., Chang J. Y., & Choi J. N. (2017). Why Reject Creative Ideas? Fear as a Driver of Implicit Bias Against Creativity. Creativity Research Journal, 29(3), 225–235.

[64] Mueller J. S., Melwani S., & Goncalo J. A. (2012). The bias against creativity: Why people desire but reject creative ideas. *Psychological science*, 23(1), 13–17. 10.1177/0956797611421018 22127366

[65] Beghetto R. A. *Dynamic perspectives on creativity*: *New directions for theory*, *research*, *and practice in education*. Springer.

[66] Corazza G. E. (2019). *The dynamic universal creativity process* *In* *Dynamic Perspectives on Creativity* (pp. 297–319). Springer, Cham.

[67] Gabora L., & Kauffman S. (2016). Toward an evolutionary-predictive foundation for creativity. *Psychonomic bulletin & review*, 23(2), 632–639. 26527351 10.3758/s13423-015-0925-1

[68] Simonton D. K. (2018). Spontaneity in evolution, learning, creativity, and free will: Spontaneous variation in four selectionist phenomena. *The Oxford Handbook of Spontaneous Thought*: *Mind-Wandering*, *Creativity*, *and Dreaming*, 113.

[69] Carruthers L., & MacLean R. (2019). The Dynamic Definition of Creativity: Implications for Creativity Assessment In *Dynamic Perspectives on Creativity* (pp. 207–223). Springer, Cham.

[70] Agnoli S., & Corazza G. E. (2019). Emotions: The spinal cord of the creative thinking process In *Dynamic Perspectives on Creativity* (pp. 47–65). Springer, Cham.

[71] Barrett L. F. (2006). Valence is a basic building block of emotional life. *Journal of Research in Personality*, 40, 35–55.

[72] Lang P. J., Greenwald M. K., Bradley M. M., & Hamm A. O. (1993). Looking at pictures: Affective, facial, visceral, and behavioral reactions. *Psychophysiology*, 30, 261–273. 8497555 10.1111/j.1469-8986.1993.tb03352.x

[73] Lang P., & Bradley M. M. (2007). The International Affective Picture System (IAPS) in the study of emotion and attention. *Handbook of emotion elicitation and assessment*, 29.

[74] Davis M. A. (2009). Understanding the relationship between mood and creativity: A meta-analysis. *Organizational behavior and human decision processes*, 108(1), 25–38.

[75] Rataj K., Nazareth D. S., & Van Der Velde F. (2018). Use a spoon as a spade?: Changes in the upper and lower alpha bands in evaluating alternate object use. Frontiers in psychology, 9, 1941 10.3389/fpsyg.2018.01941 30405471 PMC6206077

[76] Estrada C. A., Isen A. M., & Young M. J. (1997). Positive affect facilitates integration of information and decreases anchoring in reasoning among physicians. *Organizational behavior and human decision processes*, 72(1), 117–135.

[77] Isen A. M., & Patrick R. (1983). The effect of positive feelings on risk taking: When the chips are down. *Organizational behavior and human performance*, 31(2), 194–202.

[78] Russ S. W. (1993). *Affect and creativity*: *The role of affect and play in the creative process*.

[79] Fiedler K. 1988 Emotional mood, cognitive style, and behavior regulation In Fiedler K. & Forgas J. (Eds.), Affect, cognition and social behavior: 101–119. Toronto: J. Hogrefe

[80] Schwarz N., & Bless H. (1991). Happy and mindless, but sad and smart? The impact of affective states on analytic reasoning. *Emotion and social judgments*, 55–71.

[81] Murray N., Sujan H., Hirt E. R., & Sujan M. (1990). The influence of mood on categorization: A cognitive flexibility interpretation. *Journal of Personality and Social Psychology*, 59, 411–425.

[82] Berlyne D. E. (1967). Arousal and reinforcement. In Levine D (Ed.), *Nebraska Symposium on Motivation* (pp. 1–110). Lincoln: University of Nebraska Press.

[83] Dietrich A. (2004). The cognitive neuroscience of creativity. *Psychonomic bulletin & review*, 11(6), 1011–1026. 15875970 10.3758/bf03196731

[84] Easterbrook J. A. (1959). The effect of emotion on cue utilization and the organization of behavior. *Psychological review*, 66(3), 183 13658305 10.1037/h0047707

[85] Simonton D. K. (1997). Creative productivity: A predictive and explanatory model of career trajectories and landmarks. *Psychological Review*, 104, 66–89.

[86] Amabile T. M. 1996 *Creativity in context*: *Update to the social psychology of creativity*. Boulder, CO: Westview.

[87] Simonton D. K. (1984). *Genius*, *creativity*, *and leadership*: Histriometric inquiries. Boston, MA: Harvard University Press.

[88] Bower G. H. (1981). Mood and memory. *American Psychologist*, 36, 129–148. 7224324 10.1037//0003-066x.36.2.129

[89] Faul F., Erdfelder E., Lang A. G., & Buchner A. (2007). G* Power 3: A flexible statistical power analysis program for the social, behavioral, and biomedical sciences. *Behavior research methods*, 39(2), 175–191. 17695343 10.3758/bf03193146

[90] Cohen J. (1988). Statistical power analysis for the behavioral sciences (2nd ed). Hillsdale, NJ: Erlbaum.

[91] Lang P. J., Bradley M. M., and Cuthbert B. N. (2008). *International Affective Picture System (IAPS)*: *Affective Ratings of Pictures and Instruction Manual*. Technical Report A-8. Gainesville, FL: University of Florida.

[92] Mastria S., Ferrari V., & Codispoti M. (2017). Emotional picture perception: repetition effects in free-viewing and during an explicit categorization task. *Frontiers in psychology*, 8, 1001 10.3389/fpsyg.2017.01001 28725202 PMC5495866

[93] Agnoli S., Zanon M., Mastria S., Avenanti A., & Corazza G. E. (2018). Enhancing creative cognition with a rapid right-parietal neurofeedback procedure. *Neuropsychologia*. 10.1016/j.neuropsychologia.2018.02.015 29454010

[94] Wilson R. C., Guilford J. P., & Christensen P. R. (1953). The measurement of individual differences in originality. *Psychological Bulletin*, 50, 362–370. 13100527 10.1037/h0060857

[95] Silvia P. J., Winterstein B. P., Willse J. T., Barona C. M., Cram J. T., Hess K. et al (2008). Assessing creativity with divergent thinking tasks: Exploring the reliability and validity of new subjective scoring methods. *Psychology of Aesthetics*, *Creativity*, *and the Arts*, 2(2), 68.

[96] Beaty R. E., & Silvia P. J. (2012). Why do ideas get more creative across time? An executive interpretation of the serial order effect in divergent thinking tasks. *Psychology of Aesthetics*, *Creativity*, *and the Arts*, 6(4), 309.

[97] Schneider W., Eschman A., and Zuccolotto A. (2012). *E-Prime User’s Guide*. Pittsburgh: Psychology Software Tools, Inc.

[98] Lang P. J., Bradley M. M., & Cuthbert B. N. (1997). International affective picture system (IAPS): Technical manual and affective ratings. *NIMH Center for the Study of Emotion and Attention*, 39–58.

[99] Lang P. J., and Bradley M. M. (2010). Emotion and the motivational brain. *Biol*. *Psychol*. 84, 437–450. 10.1016/j.biopsycho.2009.10.007 19879918 PMC3612949

[100] Watson D., Clark L. A., & Tellegen A. (1988). Development and validation of brief measures of positive and negative affect: the PANAS scales. *Journal of personality and social psychology*, 54(6), 1063 3397865 10.1037//0022-3514.54.6.1063

[101] Dickinson A., & Dearing M. F. (1979). Appetitive-aversive interactions and inhibitory processes. *Mechanisms of learning and motivation*: *A memorial volume to Jerzy Konorski*, 203–231.

[102] Frijda N. H. (1986). *The emotions*. Cambridge University Press.

[103] Lang P. J. (1985). The cognitive psychophysiology of emotion: Fear and anxiety.

[104] Russell J. A., & Barrett L. F. (1999). Core affect, prototypical emotional episodes, and other things called emotion: dissecting the elephant. *Journal of personality and social psychology*, 76(5), 805 10353204 10.1037//0022-3514.76.5.805

[105] Russell J. A. (2003). Core affect and the psychological construction of emotion. *Psychological review*, 110(1), 145 12529060 10.1037/0033-295x.110.1.145

[106] Konorski J. (1967). Integrative activity of the brain: An interdisciplinary approach. University of Chicago Press, Chicago, IL.

[107] Wu L. (2009). *Mixed effects models for complex data*. Chapman and Hall/CRC.

[108] Greene T. R., & Noice H. (1988). Influence of positive affect upon creative thinking and problem solving in children. *Psychological reports*, 63(3), 895–898.

[109] Abele A. (1992a). Positive and negative mood influences on creativity: Evidence for asymmetrical effects. *Polish Psychological Bulletin*, 23, 203–221.

[110] Fiedler K. (2000). Toward an account of affect and cognition phenomena using the BIAS computer algorithm In Forgas J. P (Ed.), *Feeling and thinking*: *The role of affect in social cognition* (pp. 223–252). Paris: Cambridge University Press.

[111] Cramer P. (1968). *Word association*. New York: Academic Press. le, NJ: Erlbaum.

[112] Shalley C., Zhou J., & Oldham G. (2004). The effects of personal and contextual characteristics on creativity: Where should we go from here? *Journal of Management*, 30, 933–958.

[113] Vosburg S. K. (1998). Mood and the quantity and quality of ideas. *Creativity Research Journal*, 11, 315–324.

[114] Heider F. (1958). *The psychology of interpersonal relations*. New York, NY: John Wiley.

[115] Whitson J., & Galinsky A. (2008). Lacking control increases illusory pattern perception. *Science*, 322, 115–117. 10.1126/science.1159845 18832647

[116] Jackson P. W. & Messick S. (1967). The person, the product, and the response: Conceptual problems in the assessment of creativity In Kagan J. (Ed.), *Creativity and learning* (pp. 1–15). Boston, MA: Beacon Press. 10.1111/j.1467-6494.1965.tb01389.x 5826694

[117] Diedrich J., Benedek M., Jauk E., & Neubauer A. C. (2015). Are creative ideas novel and useful?. *Psychology of Aesthetics*, *Creativity*, *and the Arts*, 9(1), 35.

[118] Huang F., Tang S., Sun P., & Luo J. (2018). Neural correlates of novelty and appropriateness processing in externally induced constraint relaxation. *Neuroimage*, 172, 381–389. 10.1016/j.neuroimage.2018.01.070 29408576

[119] Mastria S., Agnoli S., Zanon M., Lubart T., & Corazza G. E. (2018). Creative brain, creative mind, creative person In *Exploring Transdisciplinarity in Art and Sciences* (pp. 3–29). Springer, Cham.

[120] Groborz M., & Necka E. (2003). Creativity and cognitive control: Explorations of generation and evaluation skills. *Creativity Research Journal*, 15(2–3), 183–197.

[121] Silvia P. J., & Phillips A. G. (2004). Self-awareness, self-evaluation, and creativity. *Personality and Social Psychology Bulletin*, 30(8), 1009–1017. 10.1177/0146167204264073 15257785

[122] Hao N., Ku Y., Liu M., Hu Y., Bodner M., Grabner R. H., & Fink A. (2016). Reflection enhances creativity: Beneficial effects of idea evaluation on idea generation. *Brain and cognition*, 103, 30–37. 10.1016/j.bandc.2016.01.005 26808451

[123] Kleinmintz O. M., Ivancovsky T., & Shamay-Tsoory S. G. (2019). The twofold model of creativity: the neural underpinnings of the generation and evaluation of creative ideas. *Current Opinion in Behavioral Sciences*, 27, 131–138.

[124] Simonton D. K. (1999a). Creativity and genius In Pervin L & John O (Eds.), *Handbook qf personality theory and research* (2nd ed, pp. 629 652~. New York: Guilford Press.

