# Supplementary material for: How does emotion influence the creativity evaluation of exogenous alternative ideas?
Source: PLoS One. 2019 Jul 5;14(7):e0219298. doi: 10.1371/journal.pone.0219298 (PMC6611619; doi:10.1371/journal.pone.0219298)
Supplement: S1 File — (DOCX) [file pone.0219298.s001.docx]

**How does emotion influence the creativity evaluation of exogenous alternative ideas?**

**Supplementary 1 - IAPS codes**

The codes for the International Affective Picture System (Lang et al., 2008) database included in the stimulus set used in the current study were as follows: Pleasant: 2530, 2550, 4597, 4599, 4601, 4604, 4607, 4608, 4609, 4611, 4616, 4619, 4622, 4623, 4624, 4625, 4626, 4640, 4641, 4643, 4645, 4647, 4649, 4650, 4651, 4652, 4653, 4656, 4658, 4659, 4660, 4664.1, 4666, 4668, 4669, 4670, 4672, 4676, 4677, 4680, 4681, 4683, 4687, 4689, 4690, 4692, 4693, 4694, 4695, 4697, 4698, 4800, 4810. Neutral: 2102, 2104, 2191, 2305, 2372, 2374, 2377, 2382, 2383, 2384, 2390, 2393, 2394, 2400, 2411, 2435, 2485, 2488, 2489, 2495, 2513, 2570, 2579, 2595, 2600, 2635, 2745.1, 2749, 2850, 4605, 5410, 7493, 9331. Unpleasant: 3000, 3010, 3015, 3016, 3017, 3030, 3051, 3053, 3059, 3060, 3061, 3062, 3063, 3064, 3068, 3069, 3071, 3080, 3100, 3101, 3102, 3103, 3110, 3120, 3130, 3131, 3140, 3150, 3160, 3168, 3170, 3180, 3181, 3185, 3190, 3191, 3195, 3212, 3213, 3225, 3261, 3266, 3301, 3400, 6200, 6210, 6212, 6213, 6220, 6231, 6242, 6243, 6244, 6250.1, 6260, 6300, 6312, 6313, 6314, 6315, 6350, 6360, 6410, 6510, 6520, 6530, 6540, 6550, 6555, 6560, 6563, 6570, 6825, 6834, 7062, 7205, 9253, 9405, 9432.
