# Supplementary material for: How does emotion influence the creativity evaluation of exogenous alternative ideas?
Source: PLoS One. 2019 Jul 5;14(7):e0219298. doi: 10.1371/journal.pone.0219298 (PMC6611619; doi:10.1371/journal.pone.0219298)
Supplement: S2 File — (DOCX) [file pone.0219298.s002.docx]

**How does emotion influence the creativity evaluation of exogenous alternative ideas?**

**Supplementary 2 - AUeT stimuli ^[[1]](#footnote-1)^ (in Italian)**

| **OBJECT** | **NonCreative use (NC)** | **Moderately Creative use (MC)** | **Highly Creative use (HC)** |
| --- | --- | --- | --- |
| accendino | come accendi sigarette | come molletta | come piscina |
| accendino | come fonte di calore | come porta ombrelli | come portafoto |
| accendino | per illuminare | come porta spazzolino | come cannuccia |
| accendino | per accendere una candela | per poggiare asciugamani | come generatore di suoni lievi |
| accendino | per accendere il fuoco | come martello | per giocare a domino |
| accendino | per accendere il gas | come specchio | come spara bolle di sapone |
| accendino | per sciogliere qualcosa | come strumento per percussioni | come tastiera colorata del pianoforte |
| appendino | per appendere abiti | come segnale stradale | come gancio per la macchina |
| appendino | come appendi oggetti | come frisbee | come chiavistello |
| appendino | come stendino | come stappa bottiglia | come compasso |
| appendino | come attaccapanni | come amo da pesca | come sagoma per un polipo |
| appendino | come porta asciugamani | come arma letale | come timpano di una casa |
| appendino | per appendere le cravatte | come strumento musicale | come manubrio della bicicletta |
| appendino | per appendere sciarpe | per creare un bracciale | come acchiappasogni |
| aspirapolvere | per aspirare | come cintura di sicurezza | come collare per cani |
| aspirapolvere | per pulire | come cuscino | come ancora |
| aspirapolvere | per pulire pavimenti | come asciugacapelli | come innaffiatoio |
| aspirapolvere | per pulire la casa | come corda | come valigia |
| aspirapolvere | per "spazzare" i tappeti | come lampada | come mazza da golf |
| aspirapolvere | per aspirare oggetti | come culla per bambini | come massaggiatore |
| aspirapolvere | per spolverare oggetti di casa | per nascondere beni preziosi | come macchina per sottovuoto |
| barattolo | come porta liquidi | per costruire due rotelle | come innaffiatoio |
| barattolo | come vasetto per i pelati | come sedia per peluches | come amplificatore di suono |
| barattolo | per conservare alimenti | come casa per uccelli | come scarpa |
| barattolo | come contenitore | come trappola | come elmo |
| barattolo | per contenere vari cibi | per disegnare circonferenze | come barchetta gallegiante |
| barattolo | per conservare qualcosa | come pentola | come microfono per modificare voce |
| barattolo | per conserve di cibo | come binocolo | come scatola del tempo |
| bicicletta | per tornare velocemente a casa | come strumento di difesa/ricatto | come griglia |
| bicicletta | come mezzo di trasporto | come scivolo | come luce appesa alla porta |
| bicicletta | per spostarsi | per giocare a calcio | per studiare il moto circolare |
| bicicletta | per fare passeggiate | come lampada da notte | come proiettore |
| bicicletta | come mezzo di locomozione | come ferma porte | come rullo tagliapasta |
| bicicletta | come veicolo | come base di un tavolo | come altalena |
| bicicletta | per trasportare oggetti | come scarpiera | per proiettare vecchi film |
| botte | come porta oggetti | come nascondiglio | come amplificatore |
| botte | come recipiente | per stirasti la schiena | come lavatoio |
| botte | come contenitore di vino | come ciondolo | come vasca |
| botte | per contenere oggetti | come portaombrelli | come zattera |
| botte | come contenitore | per fare stretching | come valigia |
| botte | per far fermentare il vino | come lampada | come altalena |
| botte | come contenitore di liquidi | come poggia piedi | come bob |
| bottiglietta | come contenitore di bevande energetiche | come saliera | come imbuto |
| bottiglietta | per bere | come tappa orecchie | come megafono |
| bottiglietta | come mini contenitore | come specchio | come massaggiatore per piedi |
| bottiglietta | come contenitore di liquidi | come lanterna | come astuccio |
| bottiglietta | come contenitore | come molletta per capelli | come burattino |
| bottiglietta | per contenere acqua | come porta candele | come poggia piedi da scrivania |
| bottiglietta | per riempirla di liquidi | come trappola per insetti | come sac a poche |
| cappello | come vestito | come presina | come barca |
| cappello | come copricapo | come ventilatore | come scarpa |
| cappello | per coprirsi dal freddo | per impagliare una sedia | come tappeto |
| cappello | per ripararsi dal sole | come porta caramelle | per filtrare l'acqua |
| cappello | come copertura dalla pioggia | come tappabuchi | per incastrare orecchini nella tela |
| cappello | per coprire la testa | come casa per insetti | come guanto |
| cappello | per coprirsi dalla pioggia | come spugna | come scolapasta |
| cucchiaio | per imboccare un bimbo | per provocare il vomito | come gamba del tavolo |
| cucchiaio | per prendere la nutella | come aiuto per cucire | per generare fuoco |
| cucchiaio | per mescolare | per dipingere | come termometro |
| cucchiaio | per prendere piccole quantità | come lancetta di un orologio | come generatore di fresco momentaneo |
| cucchiaio | per girare un fluido | per scavare | come "spillone" per i capelli |
| cucchiaio | per prendere medicine e sciroppi | per mettere ombretto sugli occhi | come calzascarpe |
| cucchiaio | per bere e mangiare | per uccidere insetti | come portalenti a contatto |
| graffetta | per segnare le pagine | come pennello | come pista per gli insetti |
| graffetta | per mettere insieme fogli | come cavatappi | come porta foto |
| graffetta | come segnalibro | per incidere sul legno | come bobina |
| graffetta | per tenere unite due cose | come mini uncino | fcome ferma lacci per scarpe |
| graffetta | come segna posto | come forchetta | come ago di una bussola |
| graffetta | come ferma carte | per fermare una cinta rotta | per girare una trottola all'interno |
| graffetta | per tenere più fogli assieme | come pircing | come gemelli delle camicie |
| guanto | per lavare i piatti | come elastico per capelli | come pinna |
| guanto | per prendere qualcosa di caldo | come tappeto | come cinturino per orologi |
| guanto | per prendere qualcosa di sporco | come tovagliolo | come acchiappamosche |
| guanto | per lavori manuali | come cresta del gallo | come alce |
| guanto | come presina | come dosatore | per creare un porta ombrelli |
| guanto | per ripararsi dal freddo | come ginocchiera | come gonna |
| guanto | per proteggere le mani | come porta penne | come extension |
| lampadina | come lampadario | come antenna | come calamita |
| lampadina | per illuminare | come bottiglia | come proiettore di immagini |
| lampadina | per creare effetti luminosi | come calice | come abito di lampadine |
| lampadina | come torcia | come contenitore | come accessorio per borse |
| lampadina | per fare luce | per misurare una circonferenza | come porta farfalle |
| lampadina | come faro | come forma per biscotti | come sostegno per rattoppare calze |
| lampadina | per illuminare un ambiente | come porta sapone | come personaggio tipo alieno |

1. Stimuli (objets images and verbal uses) were derived from the study by Agnoli et al., 2018 [↑](#footnote-ref-1)
